# Measurement of the degree of deacetylation in chitosan films by FTIR, ^1^H NMR and UV spectrophotometry

**DOI:** 10.1016/j.mex.2024.102583

**Published:** 2024-01-23

**Authors:** Dalia I. Sánchez-Machado, Jaime López-Cervantes, Ana A. Escárcega-Galaz, Olga N. Campas-Baypoli, Diana M. Martínez-Ibarra, Salvador Rascón-León

**Affiliations:** Instituto Tecnológico de Sonora, Ciudad Obregón, Sonora MX-85000, México

**Keywords:** Measurement of the degree of deacetylation in chitosan films by instrumental methods, Chitosan, Deacetylation, Films, Spectrophotometry, ^1^H NMR

## Abstract

The chitosan films were prepared from shrimp, squid, and crab to corroborate that regardless of the source of the chitosan, it was possible to measure the degree of deacetylation. In this work, the degree of deacetylation of chitosan was evaluated via UV, FTIR and ^1^H NMR spectrophotometry methodologies. Values in a range of 74 to 99% degree of deacetylation (DD) were obtained and varied depending on the method used and the source of chitosan. The spectrophotometric method is one of the most commonly used for this determination; however, it has the limitation that D-glucosamine and N-acetylglucosamine share similar wavelengths. All three methods were simple and provided rapid analysis; however, NMR, in particular, was expensive due to its equipment specifications. For this reason, its important to select the simplest method than can be routinely used.

• The simplest used technique to determine the degree of deacetylation is infrared spectroscopy.
• The degree of acetylation of chitosan is related to its physicochemical properties; its determination is an important parameter due to its association with chitosan applications in different industrial areas.
• The ^1^H NMR method is very precise and requires expensive equipment and trained personal. Thus, it cannot be used routinely to determine the degree of deacetylation.

## Graphical abstract

**Figure:**
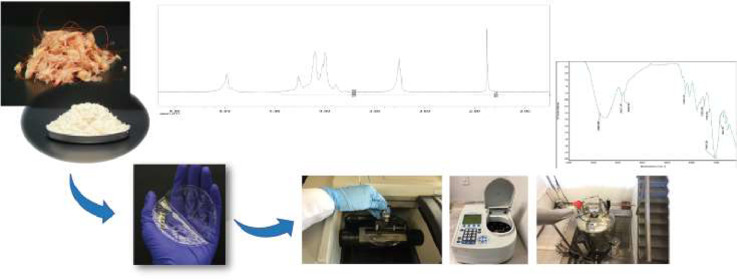

Specifications table

**Table:** 

Subject area:	Chemistry
More specific subject area:	Natural biopolymers
Name of your method:	Measurement of the degree of deacetylation in chitosan films by instrumental methods
Name and reference of original method:	D. Liu, Y. Wei, P. Yao, L. Jiang, Determination of the degree of acetylation of chitosan by UV spectrophotometry using dual standards, Carbohyd. Res. 341(6) (2006) 782-785. 10.1016/j.carres.2006.01.008
Resource availability:	NA

## Method details

### Introduction

Chitin (PubChem CID:6857375: poly-(beta-1-4) N-acetyl-D-glucosamine) is a structural homopolymer in crustaceans, insects and microorganisms. Chitosan (PubChem CID:71853) is a cationic polysaccharide produced by enzymatic or chemical deacetylation of chitin [1]. Some authors have recognized that the DD, molecular weight and viscosity of chitosan determine its applications in medicine, food preservation and agriculture [2], [3], [4], [5], [6]. The DD is a quality parameter in chitosan powder and has been related to the elongation at break and tensile strength of the chitosan films used in wound healing [7].

The DD refers to the acetyl groups attached to the polymeric chain of chitin [7]. When chitosan has more than 50% glucosamine units, it is called chitosan [8]. To determine the DD of chitosan, various methodologies have been used, including Fourier transform infrared spectroscopy (FTIR), nuclear magnetic resonance (^1^H NMR) and UV spectroscopy.

The ^1^H NMR method is very precise; however, it requires expensive equipment and trained personnel. Therefore, it cannot be used routinely to determine the DD. In the case of UV spectrophotometry, there is the problem that the D-glucosamine and N-acetylglucosamine patterns share the same absorption wavelength, causing possible interferences. On the other hand, the FTIR method has a humidity problem, which can interfere with the NH_2_ and OH groups; thus, the sample must be dry [9].

The purpose of this work is to use these methodologies for chitosan film analysis and to discuss their possible use for routine laboratory analysis by addressing the practical aspects, such as timescale and potential cost for the required instrumentation.

## Method steps

Chitosan was obtained from different sources and deacetylation methods. The conventional deacetylation method was a thermoalkaline hydrolysis with 45% NaOH at 110°C for 2 hours with constant stirring. Additionally, the microwave deacetylation method was performed with 45% NaOH at 200 W for 23 min. With both methods, the kinetic reactions were performed until the deacetylation of chitosan was reached.

### Development of chitosan films

Chitosan films were prepared by the solvent evaporation method. Specifically, 2% chitosan solutions in 1% acetic acid were prepared, and 10 ml of the polymer solution was poured into polypropylene molds and dried at 40°C. Finally, they were removed from the mold and stored in sealable bags at room temperature.

### Spectrophotometric method to measure the degree of deacetylation

The DD was determined according to the methodology of Liu et al. [10] two solutions were prepared, a standard of D-glucosamine (7.49 mM, Cg) and another of N-acetylglucosamine (0.49 mM, Ca) 0.1 N HCl; both solutions were mixed in different proportions to obtain 12 working solutions and create the calibration curve, with a concentration range for N-acetylglucosamine from 0.06 to 0.24 mM and for D-glucosamine from 0.12 to 6 mM. The absorbance readings were measured with a UV‒VIS Genesys 10 UV spectrophotometer (Ft. Madison, Iowa, USA) at 201 nm.

Specifically, 2 mg of film was weighed and diluted to 10 mL with 0.1 N HCl; after, the absorbance readings were measure with a UV‒VIS Genesys 10 UV spectrophotometer (Ft. Madison, Iowa, USA) at 201 nm. The calculations were performed with the following equation: DA = ((179.1 * A * V) – (c * m))/((k * m) - (42.1 * A * V)), where c and k correspond to the intersection point in the ordinate and to the slope of the equation of the line, A is the absorbance of the sample, V is the total volume in liters in which the sample was dissolved, and m is the amount of sample in mg. The percentage of the degree of deacetylation was calculated with the following equation: %DD = ((1-DA)*100).

### Measurement of the degree of deacetylation via FTIR

**Fig. 1:**
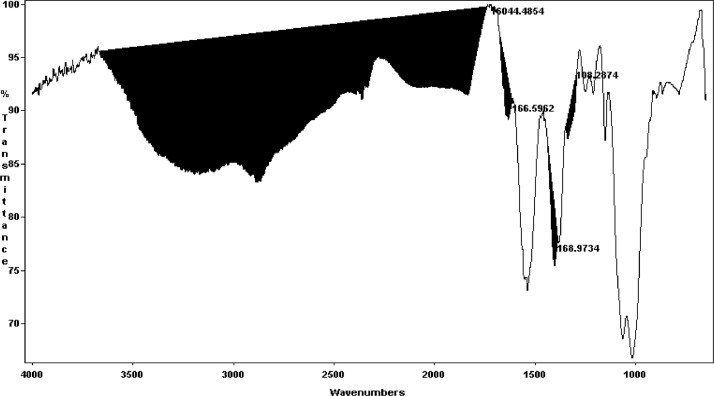
FTIR spectrum to measure the degree of deacetylation.

Infrared spectra were measured with a Mattson Genesis II FTIR. The chitosan films were placed on an ATR. The spectral resolution was 4 cm^−1^ with 16 scans in a range of 650 to 4000 cm^−1^, and the data were analyzed in Win1st software, based on the method reported by Czechowska-Biskup et al [11] with some modifications. To determine the DD, the intensities of the combination of two bands were integrated according to the equations proposed by various authors (Table 1 and Fig. 1), using the transmission (T%) mode. The measurements were performed on shrimp chitosan samples, crab and squid.

**Table 1:** Degree of deacetylation in shrimp chitosan obtained from various sources.

Method	Sample	Equation/Reference	%DD	Section
FTIR	Shrimp	DA = (A_1655_ – A_3450_) * (100/1.33) [12]	98.69 ± 0.45^e^	Different letters indicate that there is a significant difference
		DA = 31.918 * (A_1320_ – A_1420_) – 12.2 [13]	85.30 ± 1.86^c^	
		DA = ((A_1320_ – A_1420_) – 0.3822)/0.03133 [11]	74.32 ± 1.86ª	
		DA = (A_1320_ – A_1420_) *115 [14]	97.99 ± 0.69^e^	
Spectrophotometry		DA = ((179.1 * A * V) – (c * m))/((k * m) - (42.1 * A * V)) [10]	88.04 ± 0.72^d^	
^1^H NMR		DA = [(2*ACH3)/AH2-H6] * 100 [14]	80.95 ± 1.16^b^	
Spectrophotometry	Crab	DA = ((179.1 * A * V) – (c * m))/((k * m) - (42.1 * A * V) [10]	89.84 ± 1.17^a^	Different letters indicate that there is a significant difference
FTIR		DA = ((A_1320_ – A_1420_) – 0.3822)/0.03133 [11]	99.13 ± 0.15^b^	
Spectrophotometry	Squid	DA = ((179.1 * A * V) – (c * m))/((k * m) - (42.1 * A * V)) [10]	92.65 ± 1.63^a^	Different letters indicate that there is a significant difference
FTIR		DA = ((A_1320_ – A_1420_) – 0.3822)/0.03133 [11]	98.69 ± 0.32^b^	

A_1320, 1420, 1655, 3450_ correspond to the integral in the band at 1320, 1420, 1655 and 3450 cm^−1^ of the spectrum. Therefore, the degree of deacetylation was calculated by the following formula: %DD = 100 - %DA.

DA: degree of acetylation; DD: degree of deacetylation; Mean value ± standard deviation of 8 repetitions.

%DD = 100 - %DA

### Measurement of the degree of deacetylation via NMR

The DD was determined according to the methodology of Alvarenga et al. [15]. NMR spectra were measured at 70°C on a Varian Inova 17.6 T spectrometer operating at 750 MHz (proton frequency) equipped with an HCP reverse detection probe and gradients. The spectra were processed with Mestre-C v 4.9.

*Sample preparation:* Chitosan films were prepared in standard 5 mm (OD) tubes by dissolving 3 mg in 600 mL of D_2_O (%D 99.9%).

*Quantitative ^1^H NMR (^1^H spectrum).* This spectrum using the standard pulse and acquisition sequence was measured with 512 scans. The relaxation waiting time (d1) and FID (at) detection waiting time were 20 and 2 s, respectively. During the relaxation hold time, presaturation was applied for 2 s for suppression (or attenuation) of the residual undeuterated solvent (HDO) signal at ∼4.7 ppm. The spectrum was processed with apodization with an exponential function with an LB value of 2 Hz and zero filling at 64 k points. Manual phase correction and a polynomial function for baseline correction were applied. The area of selected signals from the spectrum was integrated to determine the degree of acetylation/deacetylation.

*Quantitative ^1^H NMR spectrum diffusion filter (^1^H spectrum Dfilter).* This experiment used the bipolar gradient-stimulated echo sequence (Oneshot sequence) [Pelta02]. The diffusion time Δ used was 40 ms. The duration of each bipolar gradient was 4 ms with a gradient power of 65 G/cm. The stabilization waiting time after each gradient was 0.5 ms. The spectrum was measured with 512 scans. A waiting time relaxation (d1) and y FID (at) of 20 and 2 s, respectively, were used. During the relaxation hold time, presaturation was applied for 2 s for suppression (or attenuation) of the residual undeuterated solvent (HDO) at ∼4.7 ppm. The spectrum was processed with apodization with an exponential function with an LB value of 2 Hz and zero filling at 64 k points. Manual phase correction and a polynomial function for baseline correction were applied. The area of selected signals from the spectrum was integrated to determine the degree of acetylation/deacetylation.

## Results and discussion

### Spectrophotometric method

The spectrophotometric method is one of the most widely used to determine the DD [16]. However, the D-glucosamine and N-acetylglucosamine spectra share the same absorption wavelength; thus, an interference can potentially occurs [17]. Table 1 shows the degree of deacetylation of the chitosan films according to their origin or deacetylation time.

The measured DD values for commercial chitosan vary and have been reported to be 85.82 ± 0.51% [18], 79.8% [7], and 94.1% [16]. Moreover, Aiba [19] reported a range from 69.8 to 76.7% DD, and Czechowska-Biskup et al. [11] found values from 67.83 ± 1.19 to 96.79 ± 0.34% DD.

### Fourier transform infrared spectrometry (FTIR)

The values of the DD of the chitosan films measured by FTIR are listed in Table 1. The baselines selected to calculate the DD are 1320, 1420, 1655 y 3450 cm^−1^, which have been reported by varius authors (Fig. 1).

According to the equations proposed by Barros et al. [12] and Kasaai et al. [14], the calculated DD values are high and close to 98% when applying the integration of bands 1320 (CH), 1420 (OH), 1655 (NH_2_), and 3450 (OH) cm^−1.^ Moreover, Van de Velde et al. [13] and Czechwska-Biskup et al. [11] report equations where the integration of the bands at 1320 and 1420 cm^−1^ was considered and obtained the DD values that varied from 64 to 89%. This behavior of the DD values was similar regardless of the origin of the sample.

Zhang et al. [20] reported DD values of 87%, and Chatelet et al. [6] reported a DD of 85.5% in chitosan films. Finally, Baskar et al. [3] found 85% and 83% DD in chitosans with 2 and 4 hours of hydrolysis, respectively.

### Nuclear magnetic resonance (NMR)

**Fig. 2:**
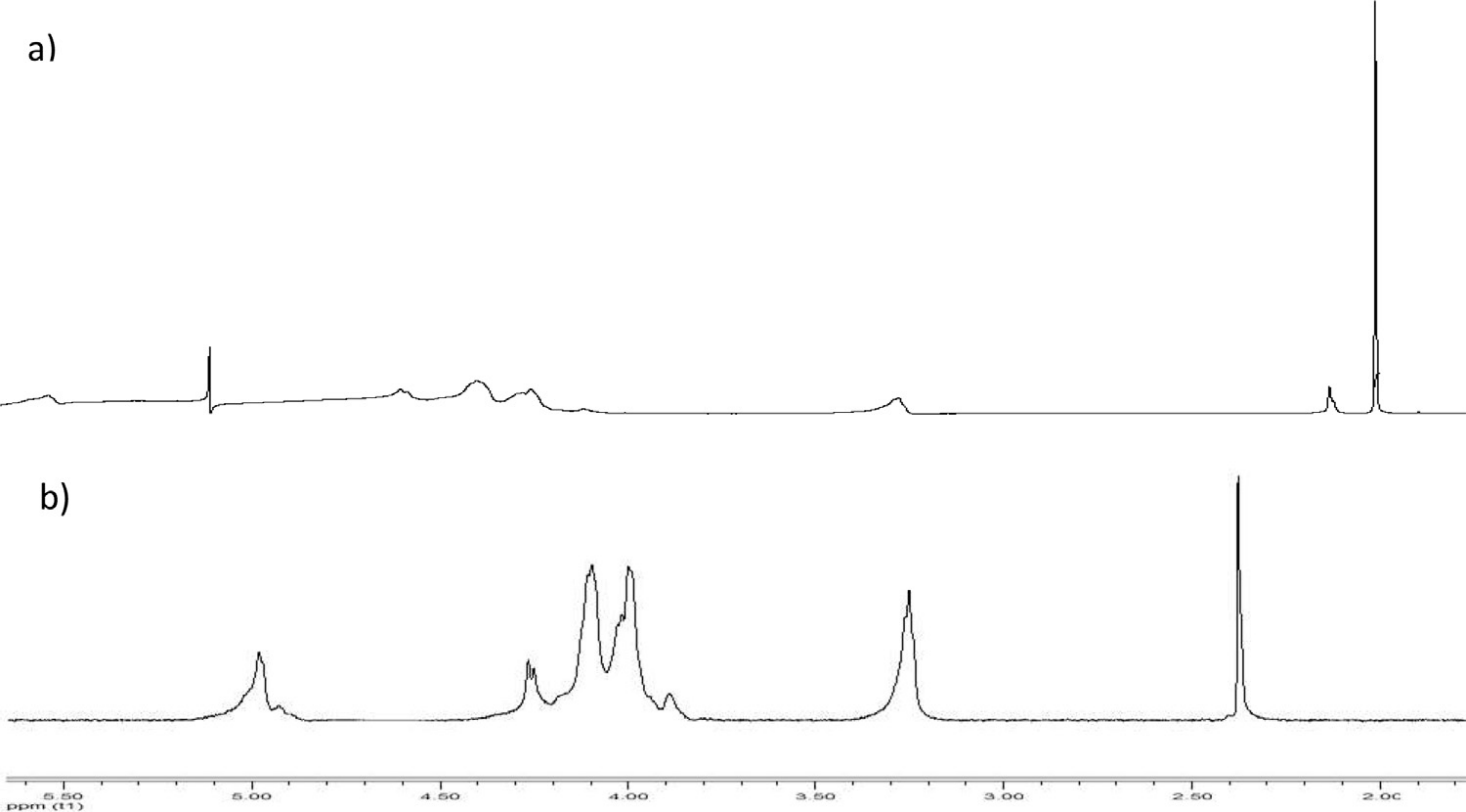
^1^H NMR spectra without a diffusion filter (a) and ^1^H NMR spectrum with a 40 ms diffusion filter of chitosan films (b).

Fig. 2(a) and (b) show the characteristic ^1^H NMR spectra of chitosan films dissolved in D_2_O. The singlet signal of the methyl protons (CH_3_) of the acetyl group was observed at 2.4 ppm. The H_2_-H_6_ protons of the pyranose ring of N-acetylglucosamine appeared as a series of unresolved multiplets in the range of 3.1 to 4.4 ppm. The anomeric proton H_1_ ring of pyranose was observed at 5.0 ppm and should have displayed a doublet multiplicity; however, this peak was not resolved due to its large width. In addition, a small peak resonating at 4.9 ppm was observed and attributed to another H_1_ anomeric proton of some minority species derived from N-acetylglucosamine. The signals found in the ^1^H NMR spectra for the characteristic functional groups of chitosan were similar to those reported by Ferandez-Megia et al. [21]. Small variations in the chemical shifts of these groups were observed and mainly attributed to the higher temperature used in the acquisition of our spectra to increase molecular mobility and thereby contribute to improving the resolution of the signals.

Based on the ^1^H NMR spectra, the degree of acetylation (%DA) and deacetylation (%DD) could be determined, and the calculations were based on the equations proposed by Alvarenga et al. [15]: %DA = [(2*ACH_3_)/AH_2_-H_6_] * 100 y %DD = 100 - %DA. where ACH_3_ corresponds to the integral of the methyl group of the acetyl and AH_2_-H_6_ corresponds to the sum of the integral of the protons H_1_-H_6_.

To obtain reliable results, a variant of the conventional quantitative proton spectrum was used to measure the quantitative proton spectrum that incorporates a diffusion filter (^1^H Dfilter). Acetate generated a peak in the ^1^H spectrum in a position that was very close to the location of the acetyl group of chitosan; thus, this acetate peak could be confused with acetyl group of chitosan. If the contribution of the acetate peak was not considered during the integration of signals from the NMR spectrum, errors in the calculation could occur. The ^1^H Dfilter experiment is an advanced way to avoid this potential problem since it is able to completely and selectively remove the acetate peak from the spectra. The ^1^H Dfilter experiment had a lower sensitivity per scan than that of the conventional 1H spectrum; however, with the experimental conditions used here, high-quality spectra were achieved with adequate sensitivity for integration (signal-to-noise ratio: SNR > 100).

Table 1 shows the values of the DD of the shrimp chitosan films from our work. The average DD value was 80.95 ± 1.16%. These results are similar to those reported by Britto et al. [22] with a range between 80% to 85%. Moreover, 84% [23] and 79.4% [20] DD values have been reported, and these were similar to those reported in our study. As the peak of the acetyl group at 2.1 ppm decreases, a higher degree of deacetylation can potentially be obtained since the acetyl functional group is removed after the deacetylation process.

A significant difference is shown between the results obtained from %DD. This is mainly because chitosan was evaluated from different sources: shrimp, crab and squid. Comparing the data between the techniques from the same source, it was observed that specifically in shrimp chitosan and using the FTIR technique, a significant difference is shown a range from 74.32 ± 1.86 to 98.69 ± 0.45, using the intensity of the characteristic N-acetylation band and the intensity of a reference band not changes with different DDs showing. In general, comparing the three techniques and the three sources of chitosans, significant differences are also shown in the data, since the values ranged between 74.32 ± 1.86 and 99.13 ± 0.15. These variations are due to the specificity of each equipment used. ^1^H NMR spectroscopy is very precise; however, it is expensive due to the need for sophisticated equipment and trained personnel. The FTIR have provides a rapid, accurate technique with a high level of precision to determinated the DD. And UV technique have been most widely used for measuring the degree of deacetylation on a routine basis.

## CRediT authorship contribution statement

**Dalia I. Sánchez-Machado:** Conceptualization, Methodology, Funding acquisition, Investigation, Writing – original draft, Writing – review & editing. **Jaime López-Cervantes:** Conceptualization, Investigation, Supervision, Writing – original draft, Writing – review & editing. **Ana A. Escárcega-Galaz:** Conceptualization, Methodology, Investigation, Writing – original draft, Writing – review & editing. **Olga N. Campas-Baypoli:** Supervision, Investigation, Writing – review & editing. **Diana M. Martínez-Ibarra:** Methodology, Supervision, Writing – original draft. **Salvador Rascón-León:** Methodology, Supervision, Writing – original draft.
